# Pelvic actinomycosis

**DOI:** 10.1093/qjmed/hcab048

**Published:** 2021-03-03

**Authors:** R Floyd, S Hunter, F Abu Saadeh, C McDonnell, P McCormick

**Affiliations:** 1 Department of Colorectal Surgery, St. James’s Hospital, Dublin 8, Ireland; 2 Department of Gynaecology, Gynaecological Cancer Care Centre, St. James’s Hospital, Dublin 8, Ireland; 3 Department of Radiology, St. James’s Hospital, Dublin 8, Ireland

## Introduction

Actinomyces is a spore-forming gram-positive facultative-anaerobe bacillus. Grocott–Gomori staining shows sulfur granules and fungus-like branched networks of hyphae leading to the name Actinomyces, ‘ray fungus’.^1^ Certain species are commensal to skin, oral, intestinal and vaginal flora. These bacteria cause actinomycosis with formation of abscesses in the mouth, lungs, or gastrointestinal tract when breaks in normal mucosal barriers occur. Actinomyces, although a benign condition, erodes weakened or damaged epithelium allowing invasion of local structures, mimicking malignancy.^2^ Predisposition to digestive tract actinomycosis can occur by disruption of gastrointestinal mucosa due to bowel surgery, inflammatory bowel conditions and perforated colonic diverticulitis.^2^

## Case report

**Figure 1.**
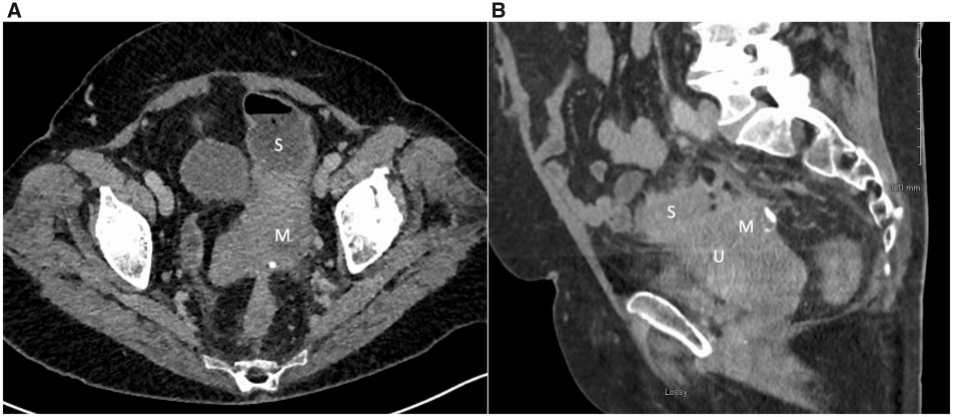
(**A**) Axial CT of the pelvis. A heterogenous mass is noted at the left side of the pelvis, with internal foci of calcification. Given the appearance of involvement of the sigmoid colon, this was initially felt to represent an inflammatory mass secondary to perforated sigmoid diverticulitis. (M—mass) (S—sigmoid colon). (**B**) Follow-up CT performed a month later demonstrates how the ill-defined mass (M) is directly involved with the fundus of the uterus (U). A loop of inflamed sigmoid colon (S) is visualized anterosuperior to the mass.

A female in her seventies presented with altered bowel habit, anorexia and abdominal discomfort with history of diverticulosis and no previous IUD use. Her abdomen was distended with left-iliac-fossa tenderness. She had raised inflammatory markers and normal tumor markers (CA125 10; CEA 1). CT-AP revealed large bowel obstruction due to a sigmoid mass (Figure 1A). She underwent a laparoscopic defunctioning sigmoid loop colostomy. A follow-up CT showed progression of the sigmoid mass and hydronephroureter (Figure 1B). Sigmoidoscopy showed sigmoid diverticula and a benign appearing stricture. Biopsies revealed mild inflammation without features of malignancy.

The initial differential diagnosis included locally perforated diverticular disease or colorectal neoplasm with the latter more likely given significant CT progression. MRI showed uterine, left adnexal and mesenteric root involvement broadening the differential to include uterine sarcoma and ovarian carcinoma. Actinomycosis was not considered in the work-up differential.

The lower gastrointestinal cancer multi-disciplinary meeting recommended surgical exploration due to non-diagnostic investigations and concern regarding malignancy. She underwent an open total abdominal hysterectomy, bilateral salpingo-oophorectomy, high anterior resection, ureteric stent insertion and small bowel resection. Her surgery was challenging due to extensive inflammation, causing dense adherence to the left pelvic sidewall. The lesion extended to the left external iliac vein and mobilization resulted in vascular injury. Specimen evaluation revealed extensive Actinomyces-induced inflammation, abscesses and fibrosis involving bowel wall, uterus, left ovary and adnexa, without evidence of endometritis, likely originating from diverticular disease.

She was commenced on intravenous benzylpenicillin and changed to intravenous ceftriaxone before discharge to facilitate outpatient antibiotics. Intravenous ceftriaxone was continued for 6 weeks post-operatively, followed by long-term oral amoxicillin for 12 months.

## Discussion

Actinomyces is a difficult diagnosis based on clinical and radiological findings but should be considered in differential diagnoses of inflammatory and particularly progressive pelvic lesions. Given the vague presenting symptoms, often mimicking malignancy, it is estimated 10% are diagnosed pre-operatively.^3^ Pre-operative diagnosis can avoid laparotomy and guide management with long-term antibiotics. In this case, laparotomy resulted in extensive resection and vascular injury necessitating 6 months anticoagulation.

While many cases of pelvic actinomycosis in women are associated with IUDs, representing 20% of actinomycosis cases reported,^3^ this case highlights the overlooked etiology of actinomycosis due to diverticular disease. Actinomyces invasion due to a breach in colonic mucosa can lead to the development of complicated actinomycosis pelvic infection.

Treatment of actinomycosis involves high-dose penicillin, recommended as intravenous therapy for 6 weeks and 12 months oral therapy to prevent recurrence.^3^ This regimen depends on the lesion size, presenting symptoms, abscess or sinus tract formation requiring definitive surgery and previous failed medical therapy.^1^ Without a pre-operative diagnosis, the lesion should be treated as a potential malignancy and excised by a surgical oncologist. Even with a pre-operative diagnosis of Actinomycosis, surgery can reduce mass effect of the lesion, increase penetration of antimicrobials, shorten treatment regimen and reduce doses required while maintaining efficacy.^4^^,^^5^

There are minimal case reports of Actinomycosis due to diverticular disease although it is likely they coexist more often than reported.^6^ The majority of cases reported presented similarly to pelvic malignancy.^3^

## Learning point for clinicians

- This is a novel atypical case of pelvic actinomycosis, likely due to underlying diverticulosis.
- Pelvic actinomycosis appears radiologically similar to progressive malignancy and should be considered in the differential diagnosis for unusual and extensive pelvic masses.
- Treatment involves surgical resection if extensive and penicillin for 1 year.

## Patient consent

Informed consent was obtained from the patient for publication of this case report.

*Conflict of interest*. None declared. 
